# Dendritic-branching angles of pyramidal neurons of the human cerebral cortex

**DOI:** 10.1007/s00429-016-1311-0

**Published:** 2016-09-30

**Authors:** Pablo Fernandez-Gonzalez, Ruth Benavides-Piccione, Ignacio Leguey, Concha Bielza, Pedro Larrañaga, Javier DeFelipe

**Affiliations:** Technical University of Madrid, Madrid, Spain

**Keywords:** Dendrite structure, Directional statistics, Branching angle distribution, Neuronal data analysis, Temporal cortex, Cortical layers

## Abstract

In this article, we analyze branching angles of the basal dendrites of pyramidal neurons of layers III and V of the human temporal cortex. For this, we use a novel probability directional statistical distribution called truncated von Mises distribution that is able to describe more accurately the dendritic-branching angles than the previous proposals. Then, we perform comparative studies using this statistical method to determine similarities and/or differences between branches and branching angles that belong to different cortical layers and regions. Using this methodology, we found that common design principles exist and govern the patterns found in the different branches that compose the basal dendrites of human pyramidal cells of the temporal cortex. However, particular differences were found between supra and infragranular cells. Furthermore, we compared the branching angles of human layer III pyramidal neurons with data obtained in the previous studies in layer III of both the rat somatosensory cortex and of several cortical areas of the mouse. Finally, we study the branching angle differences between the humans that compose our data.

**Electronic supplementary material:**

The online version of this article (doi:10.1007/s00429-016-1311-0) contains supplementary material, which is available to authorized users.

## Introduction

The design principles that govern the geometry of neurons are a major topic to those researchers interested in the generation of realistic mathematical models of neuronal morphologies. The study of pyramidal cells is of particular importance, as they are the most abundant neurons in the cortex (estimated to represent 70–80 % of the total neuronal population), where they are the main source of excitatory (glutamatergic) synapses. Furthermore, the dendritic spines of pyramidal cells constitute the main target of excitatory synapses in the cerebral cortex (DeFelipe and Farinas 1992). Thus, pyramidal cells are considered the principal building blocks of the cerebral cortex and it is thought that unravelling the morphology, connectivity, and functional organization of this type of neurons is critical for better understanding cognitive functions. There are considerable differences in the structure of pyramidal cells when considering the size and complexity of their dendritic arborization—the complexity of a dendritic arbor is evaluated as the total length of its dendritic branches along with the number and distribution of their branching points—in the density of dendritic spines on their dendritic branches and in the total number of dendritic spines. These differences are found not only between cortical areas, but also between different species, and these differences are thought to be critical for the functional specialization of the cortical areas (reviewed in Jacobs et al. 2001; Elston 2007; Elston et al. 2011; DeFelipe 2011; Eyal et al. 2014; Mohan et al. 2015).

In a previous study from our group, we found that the dendritic-branching angles of layer III pyramidal neurons in several regions of the frontal, parietal, and occipital cortex of the adult mouse follow similar principles despite the differences in the structure of these neurons in the different cortical regions examined (Bielza et al. 2014). We found that 90 % of these angles fell within a range of 20°–97°. These are similar values to the results obtained for the dendritic-branching angles of pyramidal cells from layers II–VI of the juvenile rat somatosensory cortex (angles ranged from 10° to 104°) (Leguey et al. 2016). Since the dendritic spines length is relatively short (<2 µm), it follows that the dendritic branching of pyramidal cells determines the connectivity of the pyramidal cell. Therefore, the finding is that branching angles are designed in accordance with the rules of mathematical functions and that they show common design principles that suggest a certain predictability in the synaptic connections of pyramidal cells in all the cortical areas of the mouse and rat.

In the present study, we were interested to extend these studies to the human cerebral cortex to find out if the branching angles follow similar rules using a novel branching angles data set. In particular, our aim was to try to find a statistical distribution that properly models branching angles in human pyramidal neurons and analyzes possible differences and/or similarities between branching angles in different cortical layers. More specifically, we examined layers III and V of the temporal cortex in different antero-posterior regions. We proposed the truncated von Mises distribution as the distribution to model the behavior of the dendritic-branching angles. The previous work (Bielza et al. 2014) used a different although related distribution, the von Mises distribution (Mardia 1975) as the preferred distribution to model branching angles in mice. However, the von Mises distribution alone failed to acknowledge if all the angular measurements were contained within a reduced circular interval (as it was noted in the previous study) and was forced to assume that the angles were symmetrically distributed. The truncated von Mises distribution (that is a generalization of the von Mises distribution) is able to approximate efficiently within a reduced interval non-symmetrical data, thus appearing as a more accurate analysis tool for modeling the branching angles behavior.

The rest of the paper is organized as follows. “Methods” details the different techniques chosen for the development of this work. “Results” contains the results of all the data analysis. More concretely, in “Study of branching angles by branch order” and “Study of branching angles by branch order grouped according to their maximum branch order”, we perform goodness-of-fit tests according to groups obtained by different criteria (i.e., branch order or branch order together with maximum branch order), with results that clearly improve those of the von Mises distribution. In addition, we perform hypothesis tests on different statistics related to the parameters of the distribution (such as the mean and the concentration around the mean), to further analyze the underlying patterns of the data.

In “Comparison of pairs of angles of contiguous orders”, we group the data in pairs of angles of contiguous branch orders and use the bivariate-truncated von Mises distribution as analysis tool.

In “Comparison between layer IIIPost neurons and layer VPost neurons” and “Comparison between layer IIIPost neurons and layer IIIAnt neurons”, we are interested in analyzing the differences between angular measurements that belong to different layers as well as the differences between angular measurements that belong to the same layer, but in a different region. We perform tests for a common distribution (i.e., tests that try to diagnose if two data sets could have been drawn from the same probability distribution. We will refer to them as similarity tests) between different subgroups of the data for this purpose.

In “Comparison between layer IIIAnt and IIIPost neurons and layer III neurons from mice and rats”, we analyze some results found on this study in a comparison with the data of our previous studies in mice (Bielza et al. 2014) and rats (Leguey et al. 2016). Our interest lies in finding similarities/differences of branching angles data between species, and for this, we perform tests for a common distribution of the three data sets.

Finally, “Discussion” contains the discussion of the findings and conclusions obtained throughout this study.

## Methods

### Data acquisition and preparation

Tissue was obtained from the anterolateral temporal gyri (Brodmann’s areas 21 and 38; see Garey 1994) of patients with pharmaco-resistant temporal lobe epilepsy (Department of Neurosurgery, ‘Hospital de la Princesa’, Madrid, Spain). This brain tissue was removed as part of surgical treatment of five male patients (28–48 years and mean 36.6 years) and had been used in the previous studies (Kastanauskaite et al. 2009; Arion et al. 2006; Sola et al. 2004). The five patients used in this study had normal IQs and each had a different history of medications and treatment—they were treated with a variety of anti-epileptic drugs that affect GABAergic transmission and other neurotransmitter systems. Furthermore, the disease severity was variable (with daily, weekly, or twice monthly seizures) as was the disease duration (from 10 to 29 years). However, as described below, in all the cases, the neocortical tissue used in the present study was histologically normal and without abnormal spiking activity.

In each case, video-EEG recording from bilateral foramen ovale electrodes was used to localize the epileptic focus in mesial temporal structures. Subdural recordings with a 20-electrode grid (lateral neocortex) and with a 4-electrode strip (uncus and parahippocampal) were used at the time of surgery to further identify epileptogenic regions. After surgery, the lateral temporal neocortices of all patients and the mesial temporal structures from all patients except one were available for the standard neuropathological assessment. In the latter case, most mesial structures were absorbed during surgical removal and, therefore, could not be examined. The lateral neocortices were histologically normal in all the cases. However, alterations were found in the hippocampal formations of three out of the four patients that could be examined; these three patients showed hippocampal sclerosis, whereas no apparent alterations were found in the hippocampal formation of the remaining patient. Furthermore, only neocortical tissue that showed no abnormal spiking—as characterized by normal ECoG activity—was used in this study (see Arion et al. 2006).

Surgically resected tissue was immediately immersed in cold 4 % paraformaldehyde in 0.1 M phosphate buffer, pH 7.4 (PB). After 2−3 h, the tissue was cut into small blocks (0.5 × 8 × 8 mm) which were flattened (e.g., Welker and Woolsey 1974) and post-fixed in the same fixative for 24 h at 4 °C. Horizontal sections (250 microns) were obtained using a Vibratome. By relating these sections to coronal sections, we were able to identify, using cytoarchitectural differences, the section that contained each cortical layer, allowing the subsequent injection of cells (e.g., Elston and Rosa 1997). Sections were prelabeled with 4,6-diamidino-2-phenylindole (DAPI; Sigma, St Louis, MO), and a continuous current was used to inject individual cells with Lucifer yellow (8 % in 0.1; Tris buffer, pH 7.4; LY) in cytoarchitectonically identified layers III and V of the anterolateral temporal cortex (see “Results” for further details). Neurons were injected until the individual dendrites of each cell could be traced to an abrupt end at their distal tips, and the dendritic spines were readily visible, indicating that the dendrites were completely filled. After the injection of the neurons, the sections were first processed with a rabbit antibody to Lucifer yellow produced at the Cajal Institute [1:400,000 in stock solution: 2 % BSA (A3425; Sigma); 1 % Triton X-100 (30632; BDH Chemicals); and 5 % sucrose in phosphate buffer (PB)] and then with a biotinylated donkey anti-rabbit secondary antibody (1:200 in stock solution, RPN1004; Amersham Pharmacia Biotech), followed by a biotin–horseradish peroxidase complex (1:200 in PB, RPN1051; Amersham). 3,3′-Diaminobenzidine (D8001; Sigma Chemical Co.) was used as the chromogen, allowing the visualization of the entire basal dendritic arbor of pyramidal neurons. Finally, sections were mounted in 50 % glycerol in PB.

Possible changes in the size of the sections due to processing of the tissue were evaluated by measuring the cortical surface and thickness in adjacent sections before and after intracellular injections and processing of the tissue, using Neurolucida 11.07 and StereoInvestigator 11.02.1 from MicroBrightField (MBF, VT, USA). We found no shrinkage in the surface area of the sections, and a decrease in the thickness of only approximately 7 % was observed. Therefore, no correction factors were included. Neurons were reconstructed in three dimensions using Neurolucida (MicroBrightField) as previously described in detail (for further methodological details, see Elston et al. 2001; Benavides-Piccione et al. 2006).

We refer to branch order of a branching angle as the number of branchings (including itself) that exist between the branching angle and the root of the dendrite. As an example, a branching angle with branch order 4 comes after three preceding branching angles from the root of the dendrite, which is the branch order 1. We refer to maximum branch order or tree order of a dendrite as the total amount of branch orders of a dendrite, or the branching angle at the highest order that can be found in the dendrite.

The data set included: 57, 37, and 87 cells from layer IIIAnt (1452 measurements), VPost (1328 measurements), and IIIPost (2430 measurements), respectively. More precisely, the data set for layer IIIPost contained measurements of seven branch orders (300, 477, 430, 198, 39, 5, and 3 from orders 1–7, respectively) extracted from a total of 57 neurons. The data set for layer VPost contained the measurements of eight branch orders (247, 381, 373, 226, 82, 14, 4, and 1 from orders 1–8, respectively) extracted from a total of 37 neurons. Finally, the data set for layer IIIAnt contained the measurements of seven branch orders (470, 732, 714, 375, 114, 24, and 1 from orders 1–7, respectively), extracted from a total of 87 neurons. In this data, branch orders above five suffer from a very low number of observations, and thus, we will restrict our analysis to the first five branch orders. The 3D reconstructions of these cells will be available in another publication (Benavides-Piccione, Kastanaukaite, Rojo, and DeFelipe, in preparation).

### Univariate truncated von Mises distribution

The statistical analysis of branching angles requires directional statistics, as the conventional statistics do not address well the circular properties. In this field, the von Mises distribution (Mardia 1975) is the most known distribution and the analog of the Gaussian distribution in the line. This distribution has properties, such as symmetry and positive support in all the values in a circle (0°, 360°), that are necessary simplifications of the data in many case studies. As it is found that in neuroscience, such simplifications may hinder the accuracy and reliability of the complex behaviors it studies, we propose for the first time to use the truncated von Mises distribution, and a generalization that adds two parameters that restrict the interval, where the distribution has a density greater than 0, as a step forward in better modeling the data. The truncated von Mises is defined with a four parameter probability density function:

$$ f_{\text{tvM}} (\theta ;\mu ,\kappa ,a,b) = \left\{ {\begin{array}{*{20}c} {\frac{{e^{\kappa \cos (\theta - \mu )} }}{{\int_{a}^{b} {e^{\kappa \cos (\theta - \mu )} } {\text{d}}\theta }}} & {{\text{if}}\;\;\theta \in {\mathbb{O}}_{a,b} } \\ 0 & {{\text{if}}\;\;\theta \in {\mathbb{O}}_{b,a} } \\ \end{array} } \right. $$

where *µ* ∈ ${\mathbb{O}}$ is the location parameter, *κ* > 0 the concentration parameter, ${\mathbb{O}}$ is the circular set of points, ${\mathbb{O}}$_a,b_ ⊂ ${\mathbb{O}}$ is the circular interval obtained by selecting the points in the circular path from *a* ∈ ${\mathbb{O}}$ to *b* ∈ ${\mathbb{O}}$ in the preferred direction (counterclockwise), and ${\mathbb{O}}$_b,a_ is its counterpart with respect to ${\mathbb{O}}$.

Using the truncation parameters, the distribution can present multiple shapes (strictly increasing, strictly decreasing, one global maximum, one global minimum, etc) and even not contain the mode or location parameter among the positive support. From a sample *θ*_1_, *θ*_2_,…,*θ*_n_ of angular values, the maximum likelihood estimators for parameters *a* and *b* are

$$ \begin{aligned} \hat{a} & = \hbox{min} \{ \theta_{1} , \ldots ,\theta_{n} \} \\ \hat{b} & = \hbox{max} \{ \theta_{1} , \ldots ,\theta_{n} \} . \\ \end{aligned} $$

The estimators of parameters *µ* and *κ* cannot be computed analytically, and numerical optimization techniques have to be used to approximate their value.

### Bivariate-truncated von Mises distribution

This distribution accounts for pairs of dependent angular variables. It can be used to study events that are defined by two angular measurements (*θ*_1_, *θ*_2_). It is a nine parameter distribution on the torus (${\mathbb{O}}$ × ${\mathbb{O}}$ → R), where four of the parameters correspond to that of a univariate truncated distribution for *θ*_1_ and other four correspond to that of a univariate truncated distribution for *θ*_2_ and the parameter *λ* ∈ R, that measures the correlation between *θ*_1_ and *θ*_2_, which in the circle is defined as ${\mathbb{E}}$[sin(*θ*_1_ − *µ*_1_) sin(*θ*_2_ − *µ*_2_)]. The random variable (*θ*_1_, *θ*_2_) following this distribution has the probability density function:

$$ f_{\text{btvM}} (\theta_{1} ,\theta_{2} ;{\mathbf{W}}) = \frac{{e^{{\kappa_{1} \cos (\theta_{1} - \mu_{1} ) + \kappa_{2} \cos (\theta_{2} - \mu_{2} ) + \lambda \sin (\theta_{1} - \mu_{1} )\sin (\theta_{2} - \mu_{2} )}} }}{{\int_{{a_{1} }}^{{b_{1} }} {\int_{{a_{2} }}^{{b_{2} }} {e^{{\kappa_{1} \cos (\theta_{1} - \mu_{1} ) + \kappa_{2} \cos (\theta_{2} - \mu_{2} ) + \lambda \sin (\theta_{1} - \mu_{1} )\sin (\theta_{2} - \mu_{2} )}} } } {\text{d}}\theta_{2} {\text{d}}\theta_{1} }}\quad {\text{if}}\;\;\theta_{1} \in {\mathbb{O}}_{{a_{1} ,b_{1} }} ,\theta_{2} \in {\mathbb{O}}_{{a_{2} ,b_{2} }} $$

and 0 otherwise.

**W** = {*λ*, *µ*_1_, *µ*_2_, *κ*_1_, *κ*_2_, *a*_1_, *b*_1_, *a*_2_, *b*_2_} is the parameter vector. For a sample of the form {(*θ*_*1i*_, *θ*_*2i*_) *i* = 1,…, *n*}, maximum likelihood estimators for parameters *a*_1_, *b*_1_ and *a*_2_, *b*_2_ are

$$ \begin{aligned} \hat{a}_{1} & = \hbox{min} \{ \theta_{11} , \ldots ,\theta_{1n} \} \\ \hat{b}_{1} & = \hbox{max} \{ \theta_{11} , \ldots ,\theta_{1n} \} \\ \hat{a}_{2} & = \hbox{min} \{ \theta_{21} , \ldots ,\theta_{2n} \} \\ \hat{b}_{2} & = \hbox{max} \{ \theta_{21} , \ldots ,\theta_{2n} \} . \\ \end{aligned} $$

The estimators of parameters *µ*_1_, *µ*_2_, *κ*_1_, *κ*_2,_ and *λ* cannot be computed analytically, and like in the univariate case, numerical optimization techniques have to be used for value approximation.

### Statistical tests

*Test of goodness*-*of*-*fit a univariate truncated von Mises distribution* We tested if the angular data, under different groupings, can be properly modeled with a truncated von Mises distribution. As considered in Mardia and Jupp (2000), we transformed the data *θ*_1_,…,*θ*_*n*_ by means of the angular variable *U(θ*_*i*_ *)* = *2πF(θ*_*i*_ *)*, where *F(θ)* is the probability distribution function of the truncated von Mises distribution. Then, we tested circular uniformity (i.e., the circular distribution, where every observation is equally likely to occur) using a modified Rayleigh statistic (Cordeiro and De Paula Ferrari 1991) that distributes according to a *χ*_2_^2^ distribution under the null hypothesis to obtain the final *p* value for the fit. If the data distribute following a truncated von Mises distribution, the previous transformation generated a uniform distribution from the data.

*Test of goodness*-*of*-*fit to a univariate von Mises distribution* A similar procedure is used for the von Mises distribution. The difference between both the cases is the probability distribution function *F(θ)* that is used. In this case, *F(θ)* is the probability distribution function of the von Mises distribution, and therefore, the angular variable *U(θ*_*i*_ *)* = *2πF(θ*_*i*_ *)* for this case is also different.

*Two sample tests for common distribution (similarity)* We tested the hypothesis of similarity between two data sets, i.e., if two data sets can be considered to be drawn from the same probability distribution. We used the non-parametric Watson’s two sample *U* ^2^ test (Watson 1962) that does not assume any underlying probability distribution. This test was used to perform the comparisons between layer IIIPost and layer VPost, and layer IIIAnt and layer IIIPost. In addition, it was used to perform comparisons between humans, rats, and mice (see Supplementary Tables 9, 10, and 11). Another test, the energy test (Rizzo and Szekely 2014), for the similarity of distributions outside directional statistics, was also used for the comparisons between branching angles distribution data with the “complexity” of the dendritic arbor in humans that was evaluated using the number and distribution of their branching points (i.e., total number of nodes (branch points) contained in the dendritic tree) (see Supplementary Table 15).

*Tests for mean comparison* We use Watson’s large sample (where “large” stands for samples greater or equal to 25) non-parametric test (Watson 1983) to test the null hypothesis of the same mean direction. The test does not assume any underlying probability distribution. It was used with three different subgroups of the data, as we were interested in testing if the means of the data, grouped by branchings or branchings together with maximum branch order, follow any noticeable tendency. It was additionally used for comparisons between layers IIIPost and VPost, for the comparisons of branch order 1 mean values and for the comparisons between humans, rats, and mice (see Supplementary Tables 1, 2, 4, and 12).

*Tests for the concentration comparison* Wallraff’s test for common concentration (Wallraff 1979) was useful for comparisons between layer IIIPost vs. layer VPost and layer IIIAnt vs. layer IIIPost. It is a non-parametric test with no assumptions regarding data generating distributions (see Supplementary Table 4).

*Tests of independence* We used two different tests to verify or reject the hypothesis of independence (i.e., if positive or negative significant correlations between two random variables exists). First, we used a randomized version of Rothman’s test for independence (Rothman 1971), a test that does not assume any underlying probability distribution for the two tested data sets (see Supplementary Table 8). Finally, we used a permutations tests over the λ parameter (that we previously estimated using the maximum likelihood method from the data sets) which tested the null hypothesis of *λ* = *0*

*Test*-*based diagrams* We used two different forms of visualization for the comparison of test results. The first type of diagram, the test-based diagram, was originally proposed in (Bielza et al. 2014) and consists of a space of nodes that are connected or not by edges depending on the non-rejection or rejection result of the test, respectively. In this diagram, every node that appears is pairwise tested with respect to all the other nodes. These diagrams are shown in Figs. 2d and 3. The second type of diagram, the test-based tree, is first proposed here as a form to easily visualize comparisons between two cortical brain layers or two data sets, whose data are organized in a tree-like structure that includes branch orders. It consists of trees, where the branch order in the graphic corresponds to the branch order of the conducted test. If the space between the branches is subdivided and labeled with a number, the number that labels each subdivided area indicates the maximum branch order of the data of the conducted test. Finally, the green color or red color of the area between the branches indicates the non-rejection or rejection of the hypothesis of the conducted test, respectively. These diagrams are shown in Figs. 4a, b and 5a, b.

## Results

**Fig. 1:**
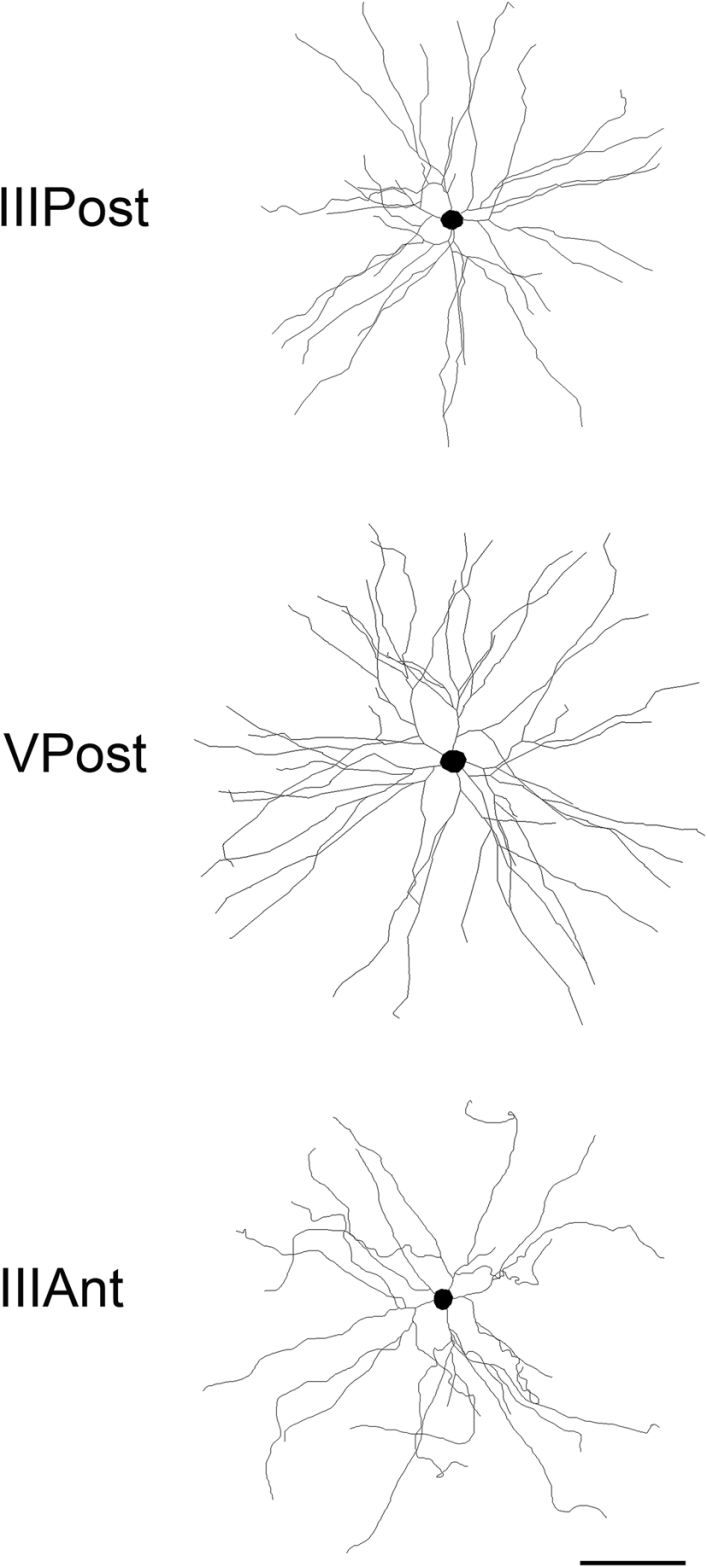
Schematic drawing examples of basal dendritic arbors of pyramidal neurons from layers III and V of the temporal cortex at a distance of 2–3 cm from the temporal pole (IIIPost and VPost, respectively) and layer III of the temporal pole proper (IIIAnt). *Scale bar* 100 µm

In the present work, a total of 181 3D reconstructed basal dendritic arbors of intra-cellularly injected cells from the human temporal cortex were included in the branch angle analysis. The cells were located in layers III and V of the temporal cortex (at a distance of 2–3 cm from the temporal pole), corresponding to Brodmann’s area 21 and in layer III of the temporal pole proper, corresponding to Brodmann’s area 38. For simplicity, we will refer to layer III anterior neurons to those located in the temporal pole as layer IIIAnt neurons, while those located at 2–3 cm will be referred as layer IIIPost and layer VPost neurons, respectively (Fig. 1).

**Fig. 2:**
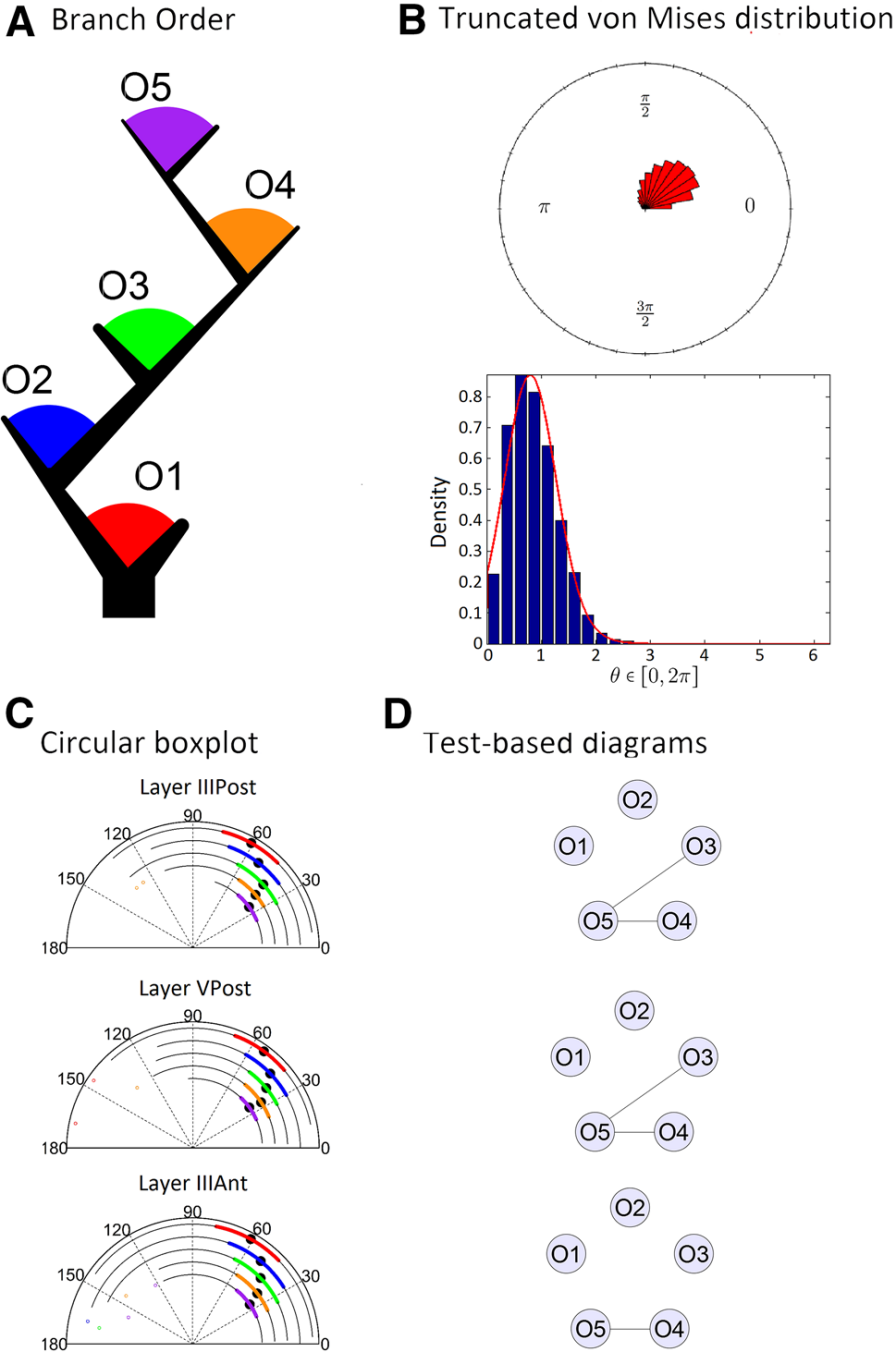
**a** Color codes for the branch orders represented in a dendritic tree. **b** Rose diagram (*top*) and truncated von Mises distribution (*bottom*) plots of the combined data of layers IIIPost, VPost, and IIIAnt. The *bars* in both the plots represent the frequency of the data. The *red curve* in the bottom plot is the estimated truncated von Mises density function. **c** *Circular boxplots* of the first five branch orders. In the different subdivisions of the *semi-circle*, we find the data summarized in different ways. The *colored curves* cover the circular interval from the lower quartile (*Q1*) to the upper quartile (*Q3*). The *longer black thin curve* covers all the values inside [Q1 + (*V*) * CIQR; Q3 − (V) * CIQR], where CIQR = Q3 − Q1 and V is 2.5 or 1.5 depending of the concentration of the data (2.5 for all our cases). The *black dot* represents the Fisher’s median statistic, and the *isolated colored dots* indicate outliers. **d** Test-based diagrams illustrating the similarity comparisons of the data groups selected in **c**. *Each node* represents a data group and two nodes are connected when the hypothesis of same probability distribution is not rejected (conversely, not connected if rejected) (see “Methods” for more details)

We first analyzed the distribution of angles of each dendritic branch order (Fig. 2a; see “Methods” for details). In general, the inspection of the rose diagrams showed that the underlying distribution for the data should be unimodal with a slight deviation from symmetry with respect to the mean (Fig. 2b). In addition, we noticed that all observations in the three data sets were contained within a circular interval that goes from 0°20^′^58^′′^ to 170°16^′^59^′′^, which covers less than half of a circle. The truncated von Mises distribution has two parameters (called *a* and *b*) that set the inferior and superior limits of the circular interval, where observations can occur, leaving a potentially non-symmetrical distribution inside. This capability makes it especially attractive for this case, and it is the justification of its choosing, together with its capability to capture unimodality.

### Study of branching angles by branch order

We compared angles of different branch orders in layers IIIPost, VPost, and IIIAnt. We will use the circular boxplots proposed in Abuzaid et al. (2012) and used in Bielza et al. (2014) as an efficient way to visualize information about the observations.

As shown in Fig. 2c, the median angular values tend to decrease, as the order increases for the three groups. This is also true for the mean angular values, decreasing as the branch order increases (see Supplementary Table 1, rows 1–10). Thus, angles in higher branch orders are smaller than those of lower branch orders. In addition, it was noticed that the angles of layer VPost are smaller in all the branch orders than the corresponding ones in layers IIIPost (see Supplementary Table 2, rows 1–5).

Regarding the concentration of the angles around the mean, angles in general showed a tendency, when compared between layers, to be similar (Supplementary Table 3). The comparison between layer IIIPost and layer IIIAnt deviated the most from these results, suggesting that the angles in layer IIIAnt may be slightly lower concentrated (see Supplementary Table 3, rows 1–5). Intuitively, a lower concentration around the mean in layer IIIAnt-branching angles implies that it is more likely to find an observation far distant from the mean in layer IIIAnt than in layer IIIPost.

Regarding the boundaries of the branching angles, the minimum angle variation (i.e., the variation of the lowest angles per bifurcations) seemed clearly lower, with a circular variance of 0.0014 radians for layer IIIPost branch orders, 0.0043 radians for layer VPost, and 0.0003 radians for layer IIIAnt, than the maximum angles variation (the variation of the highest angles per bifurcations), with a circular variance of 0.163 radians for layer IIIPost, 0.193 radians for layer VPost, and 0.038 radians for layer IIIAnt (see Supplementary Tables 5, 6, and 7 for the *a* and *b* truncation parameters that correspond to the minimum and maximum angular values).

Test-based comparisons showed that each branch order resulted significantly different from all the other branch orders except in the comparisons with the branch order 5 (Fig. 2d), which could not be rejected for branch orders 3 and 4 in layer IIIPost, branch orders 3 and 4 in layer VPost, and branch order 4 in layer IIIAnt. All the other cases presented a complete absence of links between the nodes in the test-based diagram (i.e., all the test results were rejections). Comparisons with branch order 5 may be interpreted with caution due to the small number of observations available.

The goodness-of-fit tests for the truncated von Mises distribution and the von Mises distribution revealed the modest results, with the truncated von Mises scoring 3/5 non-rejections for layer IIIPost, 3/5 non-rejections for layer VPost, and 3/5 non-rejections for layer IIIAnt (Table 1, rows 1–5). The von Mises distribution scored 3/5 non-rejections for layer IIIPost, 2/5 non-rejections for layer VPost, and 1/5 non-rejections for layer IIIAnt (Table 1, rows 1–5). These results show a slightly better performance for the truncated von Mises distribution in this case (the estimated parameter values of the truncated von Mises distribution, obtained in the tests, can be found in the Supplementary Tables 5, 6, and 7, rows 1–5).

**Table 1:** Goodness-of-fit values for the truncated von Mises distribution (TvM) and the von Mises distribution (vM) for the three data sets and the two different studies

	Layer III Post		Layer VPost		Layer IIIAnt	
	TvM	vM	TvM	vM	TvM	vM
O1	0.6268*	0.6465*	0.4353*	0.0393	0.9663*	0.6428*
O2	0.5562*	0.9626*	0.0872	0.1482*	0.0458	<0.001
O3	0.0813	0.0137	0.0370	0.0038	0.1124*	<0.001
O4	0.0688	0.0061	0.1849*	<0.001	0.2141*	<0.001
O5	0.8735*	0.8476*	0.5509*	0.1693*	0.0220	<0.001
Max1O1	0.3985*	(0.1, 0.2)*	0.7195*	<0.001	>0.95*	(0.01, 0.05)
Max2O1	0.3985*	0.0524	0.8388*	<0.001	0.4316*	0.0654
Max2O2	0.5142*	0.0575	0.4207*	0.0488	0.2275*	<0.001
Max3O1	0.8434*	0.4830*	0.4697*	0.1870*	0.3770*	0.2551*
Max3O2	0.9504*	0.7647*	0.4966*	0.0177	0.6532*	0.0172
Max3O3	0.2021*	0.2718*	0.1983*	0.0280	0.2477*	<0.001
Max4O1	0.7246*	0.7626*	0.9129*	0.3953*	0.8469*	0.6671*
Max4O2	0.4771*	0.4926*	0.8063*	0.9781*	0.2547*	0.0734
Max4O3	0.6594*	0.0079*	0.7752*	0.0010	0.2928*	<0.001
Max4O4	0.2578*	0.0213	0.2962*	<0.001	0.2030*	<0.001
Max5O1	0.7556*	0.1723*	0.9230*	0.8568*	0.9666*	0.5508*
Max5O2	0.7343*	0.3677*	0.6352*	<0.001	0.4883*	0.0622
Max5O3	0.5558*	0.1008*	0.8770*	0.0027	0.6385*	<0.001
Max5O4	0.1101*	0.0294	0.8498*	0.1210*	0.6153*	0.0205
Max5O5	0.9778*	0.0043	0.9602*	0.4863*	0.0572	<0.001

The numerical value in each cell represents the *p* value of the goodness-of-fit test. The notation OX is read as “branch order X” (for example, O3 is the branch order 3, this notation is used for the study in “Data acquisition and preparation”) and the notation MaxXOY is read as “Maximum branch order X, branch order Y” (for example, Max2O1 is the branch order 1 of dendrites with maximum branch order 2, this notation is used for the study in “Univariate truncated von Mises distribution”). If a cell contains the symbol *, it indicates that the test hypothesis was not rejected, whereas if the * symbol is missing, the opposite occurred

### Study of branching angles by branch-order-grouped according to their maximum branch order

**Fig. 3:**
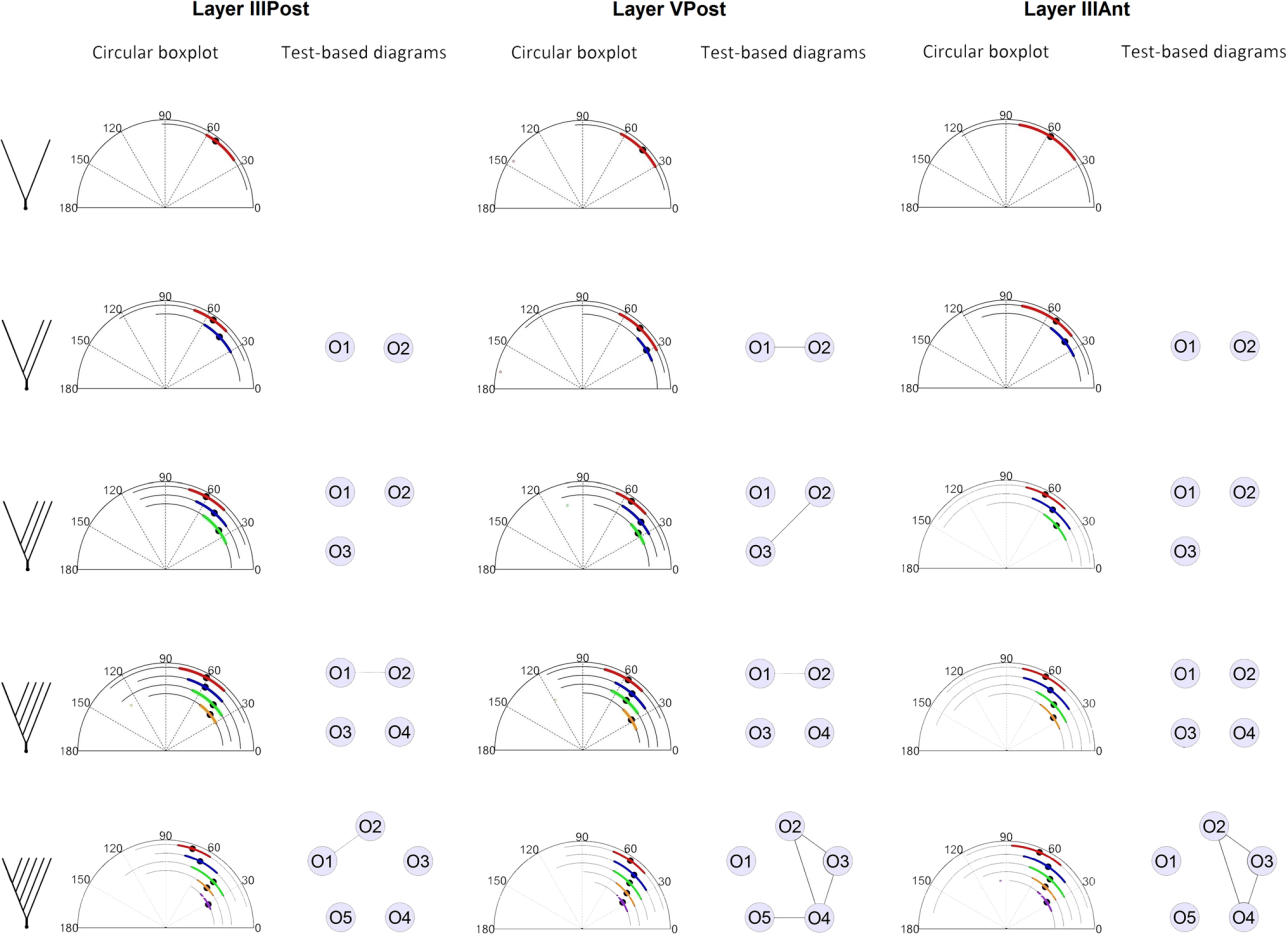
*Circular boxplots* and associated test-based diagrams coming from basal dendritic trees of pyramidal neurons grouped according to their branch complexity

Then, we compared the angles of different branch orders originating from dendritic trees of similar complexity (i.e., different dendritic trees were grouped according to their maximum branch order). The analysis showed that the previously observed tendencies for the median (Fig. 3), the tests for the mean (see Supplementary Table 1, rows 11–30 and Table 2, rows 6–20), and the concentration around the mean (see Supplementary Table 3, rows 6–20) also hold for this study.

It was found that the mean values of the first branch-order angles increase with respect to the maximum branch order (Supplementary Table 4) and this was discovered by comparing only the first branch order of dendritic trees with different maximum tree orders. In the case of the boundaries of the branching angles, it seems that the angles of the highest branch order cover a relatively small interval of angles in each maximum branch order subgroup, although it is not clear that the interval of angles decreases with the branch order as the mean does. The observed variance on the maximum angles was higher than the variance on the minimum angles in all the cases also for this study (see Supplementary Tables 5, 6, and 7, rows 6–20 for parameter values).

The similarities between branch orders resulted to be scarce, with the majority of the comparisons producing test rejections (Fig. 3). For this case, the layer with more non-rejected comparisons was layer *V* and the lowest *p* values (closer to similarity) were generally found between the first- and second-order branchings.

When performing the goodness-of-fit tests, we obtained very good results for the truncated von Mises distribution with 15/15 non-rejections for layer IIIPost, 15/15 non-rejections for layer VPost, and 14/15 non-rejections for layer IIIAnt. The von Mises distribution scored 9/15 non-rejections for layer IIIPost, 7/15 non-rejections for layer VPost, and 3/15 non-rejections for layer IIIAnt (Table 1, rows 5–19). This shows that the truncated von Mises distribution clearly outperforms the von Mises distribution in all the cases (the estimated parameter values of the truncated von Mises distribution, obtained in the tests, can be found in the Supplementary Tables 5, 6, and 7, rows 6–20). These results strengthen our belief in that grouping the data by maximum branch order and branch order is a more appropriate way to study branching angles in dendrites. It could partially shed light on why the results of grouping the data merely by branch orders are less informative.

### Comparison of pairs of angles of contiguous orders

The data were further compared in pairs of contiguous branching angles to explore the possibility that angles of the first branching may somehow influence the angles of the second branch order, using a bivariate truncated von Mises distribution. We only used the data of layer IIIAnt, since bivariate estimations require higher sample size than the univariate case. We studied if there was a measurable dependency between pairs of contiguous branch orders when fitting the distribution. We performed Rothman’s test for independence over the data of contiguous branch orders (see Supplementary Table 8). We also performed a permutation test (results not included) for *λ* = 0 in our fitted models, where *λ* is the parameter in the bivariate truncated von Mises distribution that measures the level of dependency between the two random variables (if its value is 0, both variables are considered independent). Tests results showed independence in almost all the cases

### Comparison between layer IIIPost neurons and layer VPost neurons

**Fig. 4:**
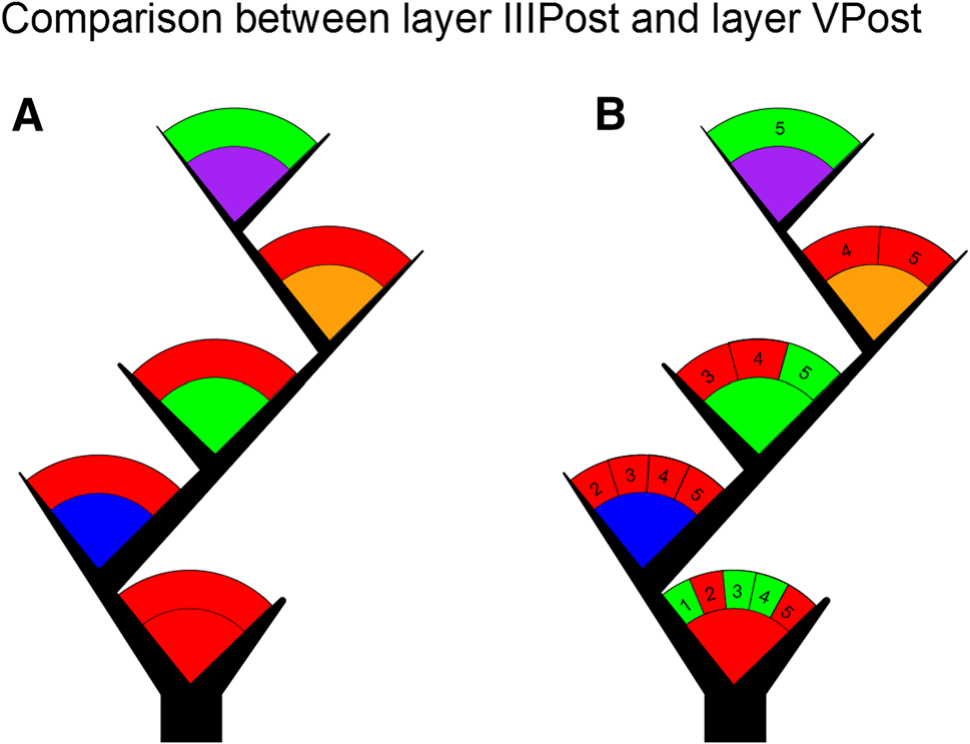
**a** Test-based tree illustrating pairwise comparisons between the branch orders in layers IIIPost and VPost. If the *arc* that appears above the branch order color code is *red*, the test produced a rejection result. If the *arc* is *green*, the result was non-rejection. **b** Comparisons of branch-order angles grouped according to their maximum branch order. The *numbers* in the *arc* above the branching color codes indicate the maximum tree order and each of the subdivisions of the arc corresponds to a test. As an example, the first branch order in the graphic shows the information of five tests performed to the first branch order of dendrites with maximum tree orders 1, 2, 3, 4, and 5

The next step was to compare angles per branch order between layers III and V. This comparison showed statistical differences with only 1/5 tests not rejected, which is the corresponding to the branch-order five comparison between the two layers (Fig. 4a, see Supplementary Table 9, rows 1–5). Then, we grouped the angles additionally by maximum branch order. In this case, we found a majority of differences (test rejections) with only 5/15 tests not rejected. More precisely, the tests that produced a non-rejection result correspond to the first branching of dendrites of maximum branch orders 1, 3, and 4, and the branch orders 3 and 5 of the dendrites of maximum branch order 5 (Fig. 4b; see Supplementary Table 9, rows 6–20). We found that, in general, angles in the first order are the most similar of all the orders compared in the same maximum branch-order group and the overall most similar (i.e., they obtained generally higher *p* values in the tests). We concluded that layers IIIPost and VPost can be considered statistically different.

### Comparison between layer IIIPost neurons and layer IIIAnt neurons

**Fig. 5:**
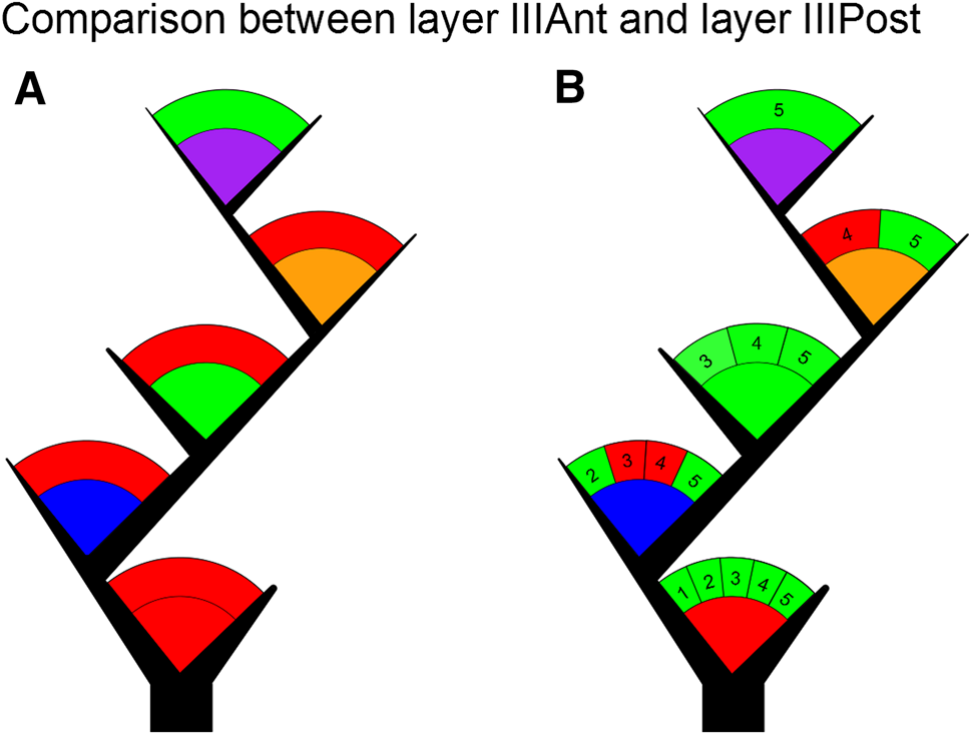
**a** Test-based tree illustrating pairwise comparisons between the branch orders in layers IIIAnt and IIIPost. If the *arc* that appears above the branch-order color code is *red*, the test produced a rejection result. If the *arc* is *green*, the result was non-rejection. **b** Comparisons of branch-order angles grouped according to their maximum branch order. The *numbers* in the *arc* above the branching color codes indicate the maximum tree order and each of the subdivisions of the *arc* corresponds to a test. As an example, the first branch order in the graphic shows the information of five tests performed to the first branch order of dendrites with maximum tree orders 1, 2, 3, 4, and 5

Similarly, we compared angles per branch order between neurons from different antero-posterior regions of the temporal cortex. We found that only 1/5 tests were not rejected (Fig. 5a; see Supplementary Table 10, rows 1–5), which corresponds to the comparison of the branch order 5. When we also grouped angles additionally by maximum branch order, and we found that non-rejections were a clear majority with 12/15 tests passed. As in the previous study in “Bivariate truncated von Mises distribution”, the angles in the first branch order could be generally considered more similar (i.e., higher *p* values), while the least similar angles were located around the branch order two, with two tests rejected for maximum branch orders 3 and 4 (Fig. 5b; see Supplementary Table 10, rows 6–20). We conclude not enough that statistical evidence was gathered to consider layers IIIAnt and IIIPost to be significantly different from each other.

### Comparison between layers IIIAnt and IIIPost neurons and layer III neurons from mice and rats

We use the data from Leguey et al. (2016) for the rat neuronal data, selecting only the layer III subset. For the mouse data, we use the data from Ballesteros-Yañez et al. (2010) selecting only the layer III subset of the wild-type mice data subset.

We first compared angular ranges eliminating 5 % of the lowest values and 5 % of the highest values. The remaining 90 % of the angular vales showed remarkable range similarities, as they ranged from 13 to 98 degrees in humans (IIIAnt and IIIPost data combined), 17°–92° in rats, and 20°–97° in mice.

However, a two sample Watson test for similarity (same distribution) between layers III neurons of human, rat, and mouse reveals significant differences between the three species (Supplementary Table 11). We further expanded our comparison between human and mouse cortical areas and performed comparisons between the layers IIIAnt and IIIPost for humans and the data for mice grouped according to seven different cortical areas, which included: primary motor cortex, secondary motor cortex, prelimbic/infralimbic cortex, primary somatosensory cortex, secondary somatosensory cortex, primary visual cortex, and secondary visual cortex. Results show in more detail the dissimilarity between both data sets with only 1/14 non-rejected tests. More specifically, we found layer IIIPost similar to primary somatosensory cortex (see Supplementary Table 12).

### Comparison between different humans under various groups of data

We now split the data into five different groups according to the different humans that generated the data. The different labels that identify them are H153, H155, H213, H263, and H264. The first comparison was between the data grouped only by different humans. The results show a majority of test rejections (9/10) with the only exception between the data of H155 and H153 (Supplementary Table 13). Subsequently, we analyze the first-order branch angle only of those groups, with the goal to locate the source of the diversity among individuals. We found that for the first branch order only, the data are remarkably different from the first study, showing a majority of non-rejections for similarity (8/10). We then continued to test other branch orders, and found that for branch order 2, the results are similar to the global study with 9/10 rejections for the same pairs of combinations, leaving the comparison of H153 and H155 as the only non-rejected case (Supplementary Table 14). Finally, we compared the number of branching angles per dendrite for all different humans, which resulted in a mixed combination between rejections (i.e., the number of nodes per dendrite does not follow a similar distribution in the comparison) and non-rejections (5/10 in both cases) (Supplementary Table 15).

## Discussion

In this article, the main objective was to analyze the branching angles of human layers III and V pyramidal neurons with the aim of trying to find a statistical distribution that properly models branching angles in human pyramidal neurons, and to find out possible differences and similarities between branching angles in different cortical layers of the temporal cortex. Furthermore, we compared the branching angles of human layer III pyramidal neurons with data obtained in the previous studies in layer III of the rat somatosensory cortex (Leguey et al. 2016) and in several cortical areas of the mouse (Bielza et al. 2014). The main conclusions are the following:

1. The truncated von Mises distribution seems to improve the results of the von Mises distribution to model branching angles, with excellent results in modeling the data.
2. Moreover, we found that branch orders nearer to the soma have the widest angles and that they gradually decrease as the branch order increases in all the groups. This was more evident when angles are selectively grouped according to the maximum branch order of their dendritic trees in all the groups, suggesting that bigger trees tend to require wider first-order angles to grow.
3. The variations between the minimum branching angles, per branch order, and maximum tree order were clearly lower than the variation of the maximum angles, which could imply that the highest branch-order angles vary less than, for example, first-order angles, which perhaps is related to the fact that the first-order angles have to allow the dendrite to grow, while the last branch-order angles are the only ones that do not have to.
4. Branch orders are shown to be statistically different from each other, which seems to be a further evidence that in the process of building a dendrite, different branch orders follow different patterns (i.e., they have to be modeled separately at least until general variation rules between branchings are found).
5. Independence tests have shown that no measurable dependency is observed between branching orders. In this direction, future work could be to consider other forms of dependency or other ways of splitting the data, where such supposed dependencies could be observed.
6. Regarding comparisons between layers III and V, angles in layer VPost were found to be clearly smaller than the angles in layer IIIPost, whereas the concentration of the angles was similar in all the cases for both the layers. The similarity tests showed that the design principles behind the formation of branching angles differ somehow between the layers IIIPost and VPost, as they can be considered statistically different. Layer IIIAnt-branching angles presented slightly lower concentrated angles than layer IIIPost. The similarity tests showed that they cannot be concluded to be statistically different by examining the data. These results are in line with the previous studies of pyramidal neurons in layer III of the mouse cerebral cortex (Bielza et al. 2014).
7. Importantly, the general rules above summarized were similar for pyramidal cells in human, rat, and mouse. Furthermore, the range of the angular-branching angles showed remarkable similarities between the three species.
8. The five individuals examined and showed significant differences in the mean branching angles among them except in one of the comparisons. However, significant differences in the branching angles for branch order 1 were only found in two of the ten comparisons, whereas for branching order 2, all were different except in one comparison. Thus, the differences between individuals are mainly due to branching angles other than for branch order 1.

Therefore, taking into consideration all these results together, we can deduce that there are common design principles that govern the geometry of dendritic-branching angles of pyramidal neurons in different layers, cortical areas, and species. These results were unexpected, as major differences in the structure of pyramidal cells are observed between these neurons in the human, rat, and mouse in terms of the size and complexity of their dendritic arborization, in the density of dendritic spines on their dendritic branches, and in the total number of dendritic spines. Thus, the present results further suggest that the branching dendritic angles do not seem to be related to the overall complexity of the dendritic arbors and number of dendritic spines, or if they are related, these differences must be due to relatively small variations in the branching angles. For example, these angles are in general wider in humans compared to rats and mice. Indeed, we found that the distribution of the branching angles of layer III pyramidal cells between the three species was statistically different in spite of the similarities of the ranges. However, when we compared the data between human layers IIIAnt and IIIPost with the data for mice grouped according to seven different cortical areas that were available (primary motor cortex, secondary motor cortex, prelimbic/infralimbic cortex, primary somatosensory cortex, secondary somatosensory cortex, primary visual cortex, and secondary visual cortex), we found that layer IIIPost was similar to primary somatosensory cortex. Thus, further similarities or differences between different species may be found by examining additional cortical regions and layers. Intuitively, the differences between the human and the mouse regarding different cortical regions would be expected, given the different functional specializations. Conversely, we do not know why there are similarities between pyramidal cells of human and mouse in areas as different as the posterior temporal cortex of humans and the primary somatosensory cortex of mouse.

Therefore, further studies are necessary to include more detailed comparisons between branch orders as the mean angle per area and the range of angles alone do not provide enough information to fully address the issue. In addition, it will be necessary to compare not only between human, rat, and mouse pyramidal neurons to try to generalize the results, but also between pyramidal cells of other species, as significant morphological differences do exist between other species (reviewed in Jacobs et al. 2001; Elston 2007; Elston et al. 2011; DeFelipe 2011; Eyal et al. 2014; Mohan et al. 2015), and it is possible that certain morphological features might be related to the dendritic-branching angles of particular branch orders in particular cortical layers, areas, or species.

Finally, the neocortex tissue of the five patients examined was histologically normal, despite the fact that these individuals were epileptic. This tissue was removed to gain access to the epileptic focus that was located in the mesial structures. In the previous studies, it has been shown that the biopsy material obtained during neurosurgical treatment for epilepsy represents an excellent opportunity to study the microanatomy of the human brain, because the resected tissue can be immediately immersed in the fixative. Thus, this tissue is lacking possible post-mortem time-induced changes that may occur at both the neurochemical and anatomical levels, which is the major problem when using brain tissue from autopsies. Certainly, this is why the quality of the immunocytochemical staining at both the light and electron microscopy levels in human biopsy material has been shown to be comparable to that obtained in experimental animals (e.g., del Río and DeFelipe 1994; Alonso-Nanclares et al. 2008). Therefore, these biopsies are of great value as for obvious ethical reasons, Therefore, these biopsies are of great value, since, for obvious ethical reasons, it is as close to a ‘normal’ sample of brain tissue as is possible to obtain for studying the human brain. However, a major drawback is that epileptic patients are heterogeneous in terms of their disease history and it is possible that the different medical characteristics of the epileptic patients (i.e., differences in the medication, severity of the disease, onset, and duration) may modify the brain tissue, but we do not have enough cases to analyze this possibility. Interestingly, the five cases examined showed significant differences in the mean branching angles among them except in the comparison between two individuals that were 28 and 41 years at the time of neurosurgery (H153 and H155, respectively). It is not known whether this represents “normal” interindividual variability or whether the differences observed were due to the different medical conditions. Nevertheless, these two “similar” cases have a rather different medical history regarding the age at onset (9 years for case H153 and 17 years for H155); the duration (19 years for case H153 and 24 years for H155); the seizure frequency (daily for H153 and weekly for H155); and the pathology observed in the mesial structures (no apparent hippocampal alterations in H153 and hippocampal sclerosis in H155). Thus, we are inclined to think that the differences between individuals may simply be due to interindividual variability. Further studies would be necessary to ascertain the range of variability between pyramidal cells of the human cerebral cortex.

## Electronic supplementary material

Below is the link to the electronic supplementary material.

Supplementary material 1 (PDF 73 kb)
