# Supplementary material for: Dendritic-branching angles of pyramidal neurons of the human cerebral cortex
Source: Brain Struct Funct. 2016 Sep 30;222(4):1847–59. doi: 10.1007/s00429-016-1311-0 (PMC5406440; doi:10.1007/s00429-016-1311-0)
Supplement: Supplementary file 1 — Supplementary material 1 (PDF 73 kb) [file 429_2016_1311_MOESM1_ESM.pdf]

Suplementary Tables

Dendritic branching angles of layers III and V of the human temporal cortex

Pablo Fernandez-Gonzalez, Ruth Benavides-Piccione, Ignacio Leguey,
Concha Bielza, Pedro Larrañaga and Javier DeFelipe

Table 1: Watson's large sample (sample size greater or equal than 25) non parametric test used for pairwise comparisons of mean angles in the three layers for the data grouped by bifurcation order and bifurcation order together with maximum bifurcation order. The value for each cell is the p−value of the test. The notation boX is read as "Bifurcation order X"(for example, bo3 is the bifurcation order 3) and the notation toXboY is read as "Maximum bifurcation order X, bifurcation order Y"(for example, to2bo1 is the bifurcation order 1 of dendrites with maximum bifurcation order 2). If a cell contains the symbol * it indicates that the null hypothesis of the test was rejected, whereas if the * symbol is missing, the opposite occured.

	Layer IIIPost	Layer VPost	Layer IIIAnt
bo1 - bo2	*0.0000	*0.0000	*0.0000
bo1 - bo3	0.2883	*0.0196	*0.0000
bo1 - bo4	*0.0000	*0.0000	*0.0000
bo1 - bo5	*0.0000	*0.0000	*0.0000
bo2 - bo3	*0.0000	*0.0000	*0.0000
bo2 - bo4	*0.0000	*0.0118	*0.0000
bo2 - bo5	*0.0000	*0.0000	*0.0000
bo3 - bo4	*0.0000	*0.0017	*0.0000
bo3 - bo5	0.2878	*0.0176	*0.0040
bo4 - bo5	*0.0084	*0.0818	> 0.95
to2bo1 - to2bo2	*0.0001	*0.0020	*0.0000
to3bo1 - to3bo2	*0.0000	*0.0001	*0.0168
to3bo1 - to3bo3	*0.0000	*0.0000	*0.0000
to3bo2 - to3bo3	*0.0000	*0.0014	*0.0000
to4bo1 - to4bo2	*0.0256	*0.0002	*0.0448
to4bo1 - to4bo3	*0.0000	*0.0000	*0.0000
to4bo1 - to4bo4	*0.0000	*0.0000	*0.0000
to4bo2 - to4bo3	*0.0021	*0.0007	*0.0000
to4bo2 - to4bo4	*0.0000	*0.0000	*0.0000
to4bo3 - to4bo4	*0.0043	*0.0022	*0.0002
to5bo1 - to5bo2	*0.0319	0.1305	*0.0248
to5bo1 - to5bo3	*0.0001	*0.0323	*0.0000
to5bo1 - to5bo4	*0.0000	*0.0000	*0.0000
to5bo1 - to5bo5	*0.0000	*0.0000	*0.0000
to5bo2 - to5bo3	*0.0127	0.4599	*0.0448
to5bo2 - to5bo4	*0.0006	*0.0066	*0.0107
to5bo2 - to5bo5	*0.0000	*0.0000	*0.0000
to5bo3 - to5bo4	0.5775	*0.0472	0.5507
to5bo3 - to5bo5	*0.0014	*0.0006	*0.0028
to5bo4 - to5bo5	*0.0016	0.1140	*0.0122

Table 2: Watson's large sample non parametric test for equal means used in comparing layer IIIPost and layer VPost. The values on each cell are the p−values of the test

	Layer IIIPost - Layer VPost
bo1	$\ast$0.0071
bo2	$\ast$0.0000
bo3	$\ast$0.0130
bo4	$\ast$0.0032
bo5	0.5223
to1bo1	$\ast$0.0000
to2bo1	0.1105
to2bo2	$\ast$0.0017
to3bo1	0.1033
to3bo2	$\ast$0.0050
t03bo3	$\ast$0.0411
to4bo1	0.4689
to4bo2	*0.0000
to4bo3	$\ast$0.0021
to4bo4	$\ast$0.0057
to5bo1	$\ast$0.0054
to5bo2	$\ast$0.0093
to5bo3	0.6187
to5bo4	$\ast$0.0757
to5bo5	0.5508

Table 3: Wallraff non parametric test for equal concentration comparing layer IIIPost and layer IIIAnt and layers IIIPost and VPost

	Layer IIIPost - Layer IIIAnt	Layer IIIPost - Layer VPost
bo1	∗0.0521	0.7703
bo2	∗0.0000	0.5488
bo3	0.2654	*0.0001
bo4	∗0.0016	0.6084
bo5	0.2618	0.7477
to1bo1	0.9330	0.5632
to2bo1	∗0.0531	*0.0452
to2bo2	0.7408	0.7010
to3bo1	0.1295	0.7376
to3bo2	0.1114	0.7600
t03bo3	0.6051	*0.0002
to4bo1	0.8748	0.7864
to4bo2	*0.0270	0.4784
to4bo3	0.4584	*0.0005
to4bo4	0.1186	0.5818
to5bo1	0.5484	0.9394
to5bo2	0.1197	0.2768
to5bo3	0.8025	0.4423
to5bo4	∗0.0773	0.2123
to5bo5	0.1284	0.7616

Table 4: Watson's large sample (sample size greater or equal than 25) non parametric test used for pairwise comparisons of mean angles for the first bifurcation order in the data grouped by bifurcation order together with maximum bifurcation order. The value inside each cell is the p-value of the test.

	Layer IIIPost	Layer VPost	Layer IIIAnt
to1bo1 - to2bo1	0.2078	0.8436	0.8117
to1bo1 - to3bo1	*0.0495	0.1349	0.8364
to1bo1 - to4bo1	*0.03199	*0.0179	0.4910
to1bo1 - to5bo1	*0.0040	0.1780	0.1623
to2bo1 - to3bo1	0.3726	0.2121	0.5380
to2bo1 - to4bo1	0.1721	*0.0264	0.2119
to2bo1 - to5bo1	*0.0171	0.2900	*0.04122
to3bo1 - to4bo1	0.5207	0.1726	0.4641
to3bo1 - to5bo1	*0.0456	0.9792	*0.0840
to4bo1 - to5bo1	0.1546	0.2294	0.2260

Table 5: Parameter values for the truncated von Mises distribution in layer IIIPost data (for a description of each parameter, see subsection 4.3 of the main article)

	μ	κ	a	b
bo1	1.0696	6.4688	0.1311	2.2940
bo2	0.9168	6.1540	0.0308	1.9713
bo3	0.7325	4.7578	0.0267	2.2942
bo4	0.7151	6.2501	0.0483	2.3235
bo5	0.5996	8.3522	0.0606	1.2433
to1bo1	0.8659	3.2636	0.2158	1.6066
to2bo1	0.9372	4.8716	0.3415	2.1492
to2bo2	0.7254	5.1648	0.0990	1.6969
to3bo1	1.0580	6.4654	0.3071	1.9694
to3bo2	0.8616	6.7724	0.1515	1.9156
to3bo3	0.6457	5.7175	0.0267	1.872
to4bo1	1.0642	5.3604	0.3438	2.2940
to4bo2	0.9918	5.5867	0.0308	1.9568
to4bo3	0.7380	4.0043	0.1465	2.2942
to4bo4	0.6628	6.4653	0.0974	2.2291
to5bo1	1.3199	3.7438	0.2564	1.9437
to5bo2	1.0540	5.5045	0.3603	1.7144
to5bo3	0.7412	3.3566	0.0407	2.2361
to5bo4	0.752	5.3046	0.0483	2.3235
to5bo5	0.6247	4.6047	0.0606	1.0235

Table 6: Parameter values for the truncated von Mises distribution in layer VPost data.

	μ	κ	a	b
bo1	0.9302	4.9821	0.2000	2.9300
bo2	0.7710	6.2926	0.0071	1.9069
bo3	0.6877	6.8251	0.0451	1.9689
bo4	0.5662	5.7993	0.0551	2.3169
bo5	0.6051	7.7482	0.0717	1.6360
to1bo1	0.2202	1.9266	0.2240	2.5447
to2bo1	0.5441	7.1456	0.2000	2.9362
to2bo2	0.4900	5.9687	0.071	1.5645
to3bo1	0.9752	6.3212	0.2337	1.7589
to3bo2	0.6490	5.6996	0.1712	1.8623
to3bo3	0.5663	8.5728	0.0451	1.8113
to4bo1	0.9995	5.0227	0.3934	2.0146
to4bo2	0.8527	6.6955	0.0442	1.9069
to4bo3	0.6167	6.0677	0.1962	1.8297
to4bo4	0.372	4.3621	0.1352	2.0732
to5bo1	0.9712	7.4108	0.2379	1.7823
to5bo2	0.5871	2.2126	0.2350	1.7862
to5bo3	0.6528	3.4122	0.1404	1.9689
to5bo4	0.5912	5.1655	0.0331	2.3169
to5bo5	0.5704	9.5889	0.0717	1.3012

Table 7: Parameter values for the truncated von Mises distribution in layer IIIAnt data

	μ	κ	a	b
bo1	1.0515	4.8347	0.0605	2.5154
bo2	0.8457	3.3175	0.0061	2.9288
bo3	0.6922	3.5262	0.0394	2.9720
bo4	0.6171	4.1737	0.0330	2.5145
bo5	0.4562	3.1667	0.0217	2.7488
to1bo1	0.9812	1.7861	0.0605	2.1251
to2bo1	0.9469	3.1029	0.1662	2.1031
to2bo2	0.5421	3.9988	0.0917	1.8966
to3bo1	1.0343	4.7488	0.068	2.5154
to3bo2	0.8382	3.2134	0.029	2.5618
to3bo3	0.5924	3.6275	0.0999	1.8695
to4bo1	1.0839	5.3113	0.0882	2.3704
to4bo2	0.9287	3.32	0.0061	2.7917
to4bo3	0.6876	3.2789	0.0394	2.4845
to4bo4	0.5703	4.7126	0.0330	1.8994
to5bo1	1.1826	4.3745	0.2526	2.0628
to5bo2	0.9157	3.2046	0.0703	2.9288
to5bo3	0.6350	2.7122	0.1396	2.972
to5bo4	0.7334	3.6254	0.0432	2.5145
to5bo5	0.4241	2.9514	0.0217	2.1359

Table 8: Rothman's test for independence between pairs of continuous bifurcation order samples grouped by maximum bifurcation order in layer IIIAnt.

	Layer IIIAnt
to2bo1 - to2bo2	0.422
to3bo1 - to3bo2	0.549
to3bo3 - to3bo2	0.5540
to4bo1 - to4bo2	0.3030
to4bo2 - to4bo3	0.4880
to4bo3 - to4bo2	*0.004
to5bo1 - to5bo2	*0.001
to5bo2 - to5bo3	0.259
to5bo3 - to5bo4	0.960
to5bo4 - to5bo4	0.247

Table 9: Two sample Watson test for similarity (same distribution) between layers IIIPost and VPost. The value inside each cell is an interval, according to common significance levels, that contains the $p-$value of the test.

	Layer IIIPost - Layer VPost
bo1	$*(0.05, 0.1)$
bo2	$* < 0.001$
bo3	$*(0.01, 0.05)$
bo4	$*(0.001, 0.01)$
bo5	> 0.1
to1b1	> 0.1
to2b1	$*(0.01, 0.05)$
to2b2	$*(0.01, 0.05)$
to3b1	> 0.1
to3b2	$*(0.01, 0.05)$
t03b3	$*(0.01, 0.05)$
to4b1	> 0.1
to4b2	*(0.05,0.1)
to4b3	$*(0.01, 0.05)$
to4b4	$*(0.01, 0.05)$
to5b1	$*(0.01, 0.05)$
to5b2	$*(0.001, 0.01)$
to5b3	> 0.1
to5b4	$*(0.05, 0.1)$
to5b5	> 0.1

Table 10: Two sample Watson test for similarity (same distribution) between layers IIIAnt and IIIPost. The value inside each cell is an interval, according to common significance levels, that contains the p–value of the test.

	Layer IIIPost - Layer IIIAnt
bo1	$*(0.01, 0.05)$
bo2	$* < 0.001$
bo3	$* < 0.001$
bo4	$*(0.01, 0.05)$
bo5	> 0.1
to1b1	> 0.1
to2b1	> 0.1
to2b2	> 0.1
to3b1	> 0.1
to3b2	$*(0, 001, 0.01)$
t03b3	> 0.1
to4b1	> 0.1
to4b2	*(0.001, 0.01)
to4b3	> 0.1
to4b4	$*(0.05, 0.1)$
to5b1	> 0.1
to5b2	> 0.1
to5b3	> 0.1
to5b4	> 0.1
to5b5	> 0.1

Table 11: Two sample Watson test for similarity (same distribution) between layer III neurons of human, rat and mouse. The value of each cell is an interval, according to common significance levels, that contains the p−value of the test.

Human - Mouse - Rat	
Human-Rat	$* < 0.001$
Human-Mouse	$* < 0.001$
Rat-Mice	$* < 0.001$

Table 12: Two sample Watson test for similarity (same distribution) between the data of the layer III of the cortex in humans and data of layer III of different cortical areas in mice. The names M1,M2,PrL,S1,S2,V1 and V2, stand, respectively for: primary motor cortex, secondary motor cortex, prelimbic/infralimbic cortex, primary somatosensory cortex, secondary somatosensory cortex, primary visual cortex and secondary visual cortex

Human - Mouse	
Layer IIIAnt - M1	$* < 0.001$
Layer IIIAnt - M2	$* < 0.001$
Layer IIIAnt - PrL	$* < 0.001$
Layer IIIAnt - S1	$* < 0.001$
Layer IIIAnt - S2	$* < 0.001$
Layer IIIAnt - V1	$* < 0.001$
Layer IIIAnt - V2	$* < 0.001$
Layer IIIPost - M1	$* < 0.001$
Layer IIIPost - M2	$* < 0.001$
Layer IIIPost - PrL	$* < 0.001$
Layer IIIPost - S1	> 0.1
Layer IIIPost - S2	$* < 0.001$
Layer IIIPost - V1	$* < 0.001$
Layer IIIPost - V2	$*(0.01, 0.05)$

Table 13: Two sample Watson test for similarity (same distribution) between the data of the different humans that provided the samples. Each human is identified by an id of the form HXYZ, where X,Y and Z are digits.

H153 - H155 - H213 - H263 - H264	
H153 - H155	> 0.1
H153 - H213	$* < 0.001$
H153 - H263	$* < 0.001$
H153 - H264	$* < 0.001$
H155 - H213	$* < 0.001$
H155 - H263	$*(0.001, 0.01)$
H155 - H264	$*(0.001, 0.01)$
H213 - H263	$* < 0.001$
H213 - H264	$*(0.001, 0.01)$
H263 - H264	$*(0.05, 0.1)$

Table 14: Two sample Watson test for similarity (same distribution) between the data, grouped by branch orders 1-2, of the different humans that provided the samples.

H153 - H155 - H213 - H263 - H264	
H153bo1 - H155bo1	> 0.1
H153bo1 - H213bo1	$*(0.001, 0.1)$
H153bo1 - H263bo1	> 0.1
H153bo1 - H264bo1	> 0.1
H155bo1 - H213bo1	$*(0.05, 0.1)$
H155bo1 - H263bo1	> 0.1
H155bo1 - H264bo1	> 0.1
H213bo1 - H263bo1	> 0.1
H213bo1 - H264bo1	> 0.1
H263bo1 - H264bo1	> 0.1
H153bo2 - H155bo2	> 0.1
H153bo2 - H213bo2	$* < 0.0001$
H153bo2 - H263bo2	$* < 0.0001$
H153bo2 - H264bo2	$*(0.001, 0.01)$
H155bo2 - H213bo2	$* < 0.0001$
H155bo2 - H263bo2	$* < 0.0001$
H155bo2 - H264bo2	$*(0.05, 0.1)$
H213bo2 - H263bo2	$*(0.01, 0.05)$
H213bo2 - H264bo2	$*(0.05, 0.1)$
H263bo2 - H264bo2	$*(0.001, 0.01)$

Table 15: Energy test for similarity (same distribution) between the data of the number of nodes of the dendrites of the different humans that provided the samples.

H153 - H155 - H213 - H263 - H264	
H153 - H155	$\star$0.0041
H153 - H213	$\star$0.0043
H153 - H263	$\star$0.0369
H153 - H264	0.2258
H155 - H213	0.2836
H155 - H263	0.2924
H155 - H264	$\star$0.0769
H213 - H263	0.8842
H213 - H264	$\star$0.0307
H263 - H264	0.1033
